# Publisher Correction: MBNL1 regulates essential alternative RNA splicing patterns in MLL-rearranged leukemia

**DOI:** 10.1038/s41467-020-17210-8

**Published:** 2020-07-07

**Authors:** Svetlana S. Itskovich, Arun Gurunathan, Jason Clark, Matthew Burwinkel, Mark Wunderlich, Mikaela R. Berger, Aishwarya Kulkarni, Kashish Chetal, Meenakshi Venkatasubramanian, Nathan Salomonis, Ashish R. Kumar, Lynn H. Lee

**Affiliations:** 1 Division of Bone Marrow Transplantation and Immune Deficiency, Cincinnati Children’s Hospital Medical Center, Cincinnati, OH 45229 USA; 2 Cancer and Blood Diseases Institute, Cincinnati Children’s Hospital Medical Center, Cincinnati, OH 45229 USA; 3 Division of Experimental Hematology and Cancer Biology, Cincinnati Children’s Hospital Medical Center, Cincinnati, OH 45229 USA; 4 College of Medicine, University of Cincinnati School of Medicine, Cincinnati, OH 45267 USA; 5 Department of Electrical Engineering and Computer Science, University of Cincinnati, Cincinnati, OH 45221 USA; 6 Division of Biomedical Informatics, Cincinnati Children’s Hospital Medical Center, Cincinnati, OH 45229 USA; 7 Department of Pediatrics, University of Cincinnati School of Medicine, Cincinnati, OH 45229 USA; 8 Division of Oncology, Cincinnati Children’s Hospital Medical Center, Cincinnati, OH 45229 USA

**Keywords:** Cancer, Haematological cancer, Leukaemia

Correction to: *Nature Communications* 10.1038/s41467-020-15733-8, published online 12 May 2020.

The original version of this Article contained an error in Fig. 6. In Fig. 6d the labels for the lower circles of the Venn diagram were exchanged. The left lower circle was labelled “MOLM13 cell line MBNL1 sh65” instead of the correct “MOLM13 cell line MBNL1 sh64”. The right circle was labelled “MOLM13 cell line MBNL1 sh64” instead of the correct “MOLM13 cell line MBNL1 sh65”.

In addition, in the original version of this Article reference 46 was duplicated from reference 62. Reference 62 has been deleted and the following references renumbered accordingly.

These errors have been corrected in the PDF and HTML versions of the Article.

