# Supplementary material for: MBNL1 regulates essential alternative RNA splicing patterns in MLL-rearranged leukemia
Source: Nat Commun. 2020 May 12;11:2369. doi: 10.1038/s41467-020-15733-8 (PMC7217953; doi:10.1038/s41467-020-15733-8)
Supplement: Supplementary file 1 — Supplementary Information [file 41467_2020_15733_MOESM1_ESM.pdf]

MBNL1 regulates essential alternative RNA splicing patterns in MLL-rearranged leukemia

Itskovich and Gurunathan, et al.

Supplementary Information

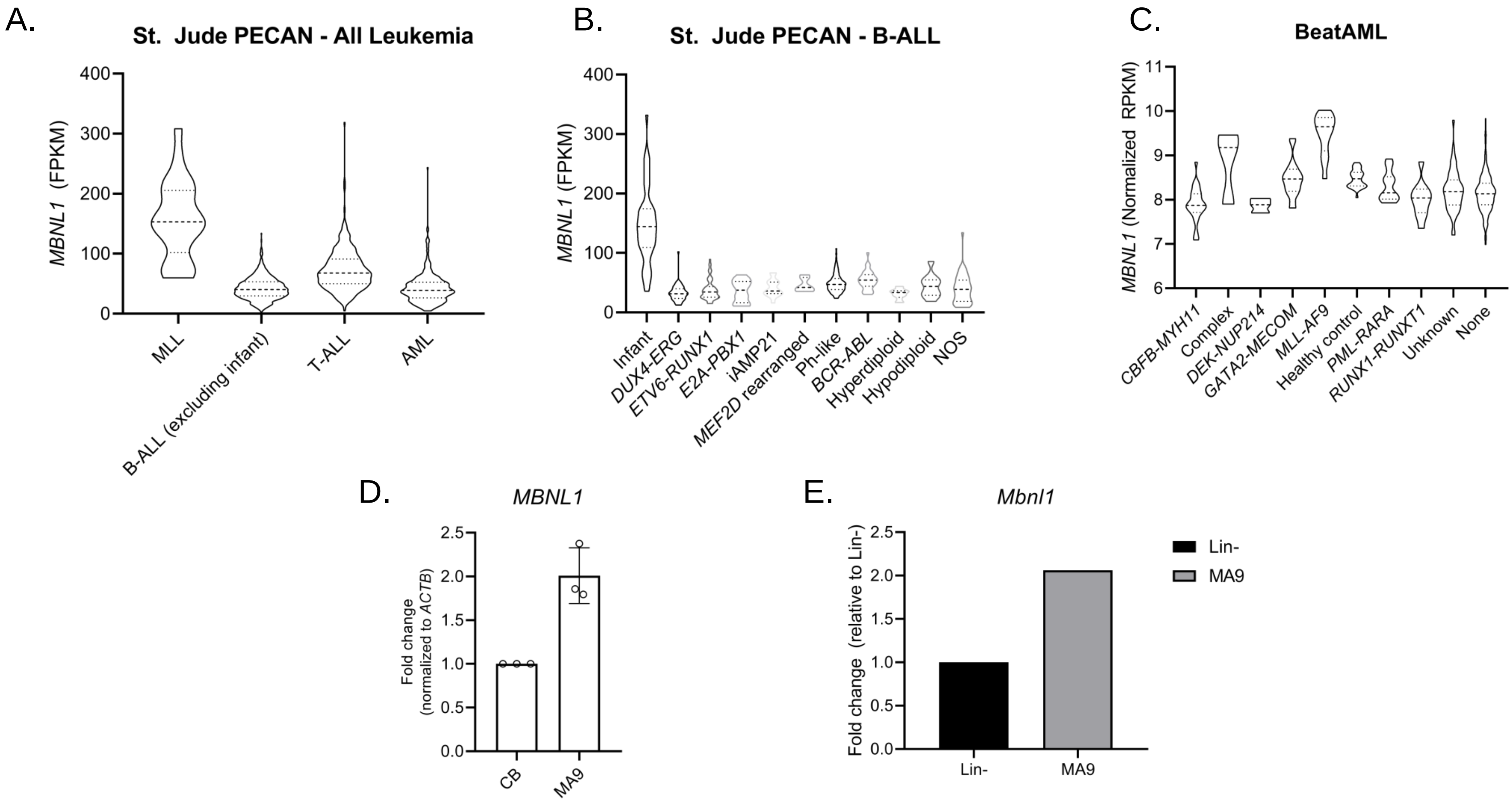

Supplementary Figure 1: *MBNL1* expression in pediatric leukemia samples stratified by A) lineage (n = 1080; *MLL* n = 24), and B) within pre-B ALL by dominant genetic alteration[1-5] (n = 403; infant n = 42) accessed via the St. Jude Cloud. C) Adult primary AML samples[6] with normalized RPKM for *MBNL1* (n = 483; *MLL-AF9* n = 13). Thick dashed line represents median; thin dashed lines represent quartiles. D) qRT-PCR analysis of MBNL1 expression in CD34+ cord blood cells (CB) before and after transformation with MLL-AF9 (MA9) oncogene. Bars show mean ± SD. Data is from 3 biological replicates. E) Representative qRT-PCR analysis of *Mbnl1* expression in Lin- mouse bone marrow expression before and after transformation with MLL-AF9 (MA9) oncogene.

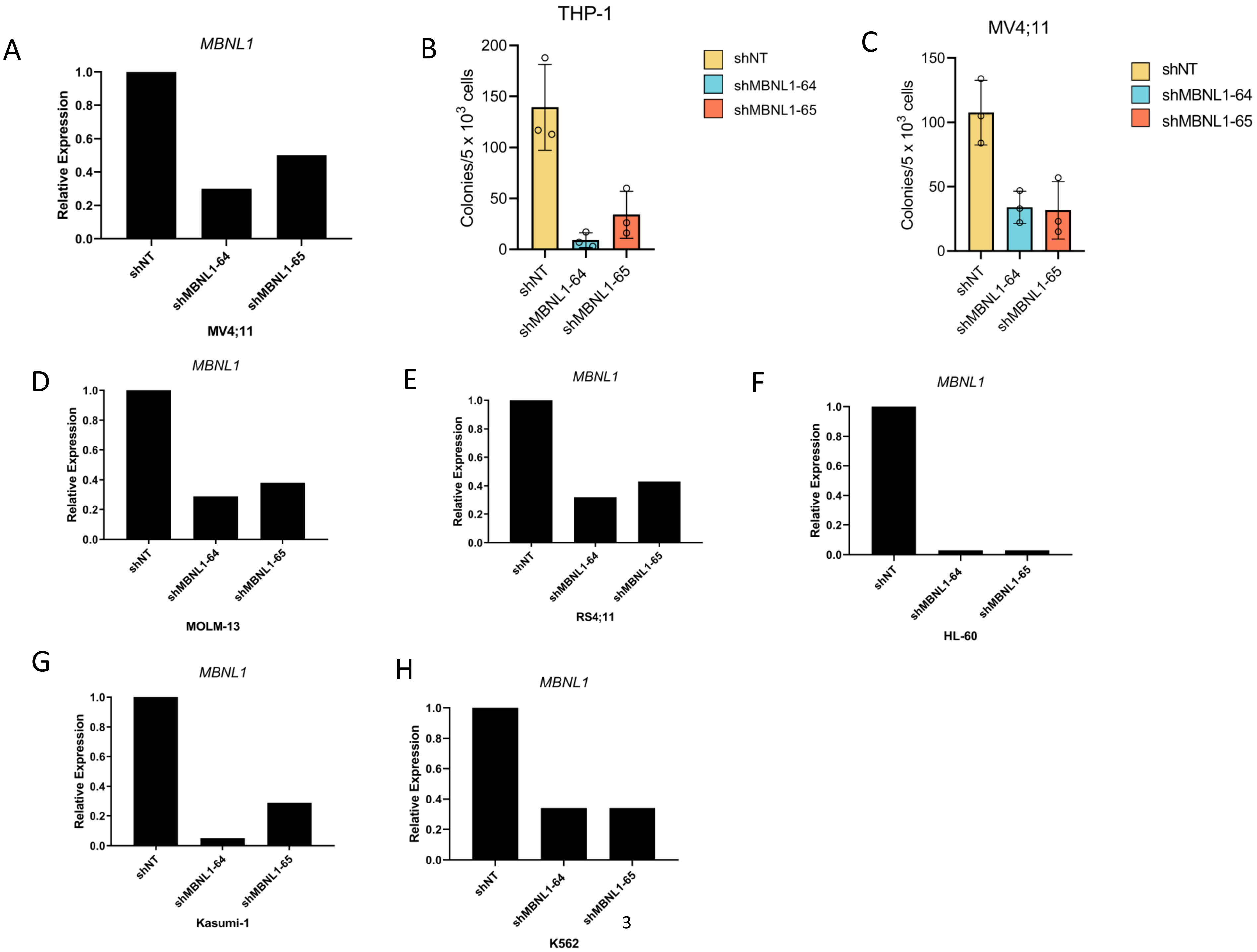
A
MBNL1
Relative Expression
1.0
0.8
0.6
0.4
0.2
0.0
shNT
shMBNL1-64
shMBNL1-65
MV4;11
B
THP-1
Colonies/5 x 10^3 cells
200
150
100
50
0
shNT
shMBNL1-64
shMBNL1-65
C
MV4;11
Colonies/5 x 10^3 cells
150
100
50
0
shNT
shMBNL1-64
shMBNL1-65
D
MBNL1
Relative Expression
MOLM-13
E
MBNL1
Relative Expression
RS4;11
F
MBNL1
Relative Expression
HL-60
G
MBNL1
Relative Expression
Kasumi-1
H
MBNL1
Relative Expression
K562

Supplementary Figure 2. Additional *in vitro MBNL1* knockdown studies. (A). Knockdown of *MBNL1* was performed in MV4;11 cells using different shMBNL1 RNAs (shMBNL1-64 and shMBNL1-65 are listed). After transduction, the levels of *MBNL1* expression were tested by qRT-PCR. The graph represents relative expression of *MBNL1* normalized to *β-ACTIN*. **(B-C)**. CFU activity of THP-1 (B.) and MV4;11 (C.) cells upon *MBNL1* knockdown. Bars show mean ± SD. Data is from 3 biological replicates. **(D-F)**. qRT-PCR data confirming *MBNL1* knockdown with shMBNL1-64 and shMBNL1-65 in MOLM13 (D.), RS4;11 (E.), HL-60 (F.), Kasumi-1 (G.), and K562 (H.) cells in the *in vitro* growth assay shown in Figure 2B-2G. The graphs represent relative expression of *MBNL1* normalized to *ACTB*.

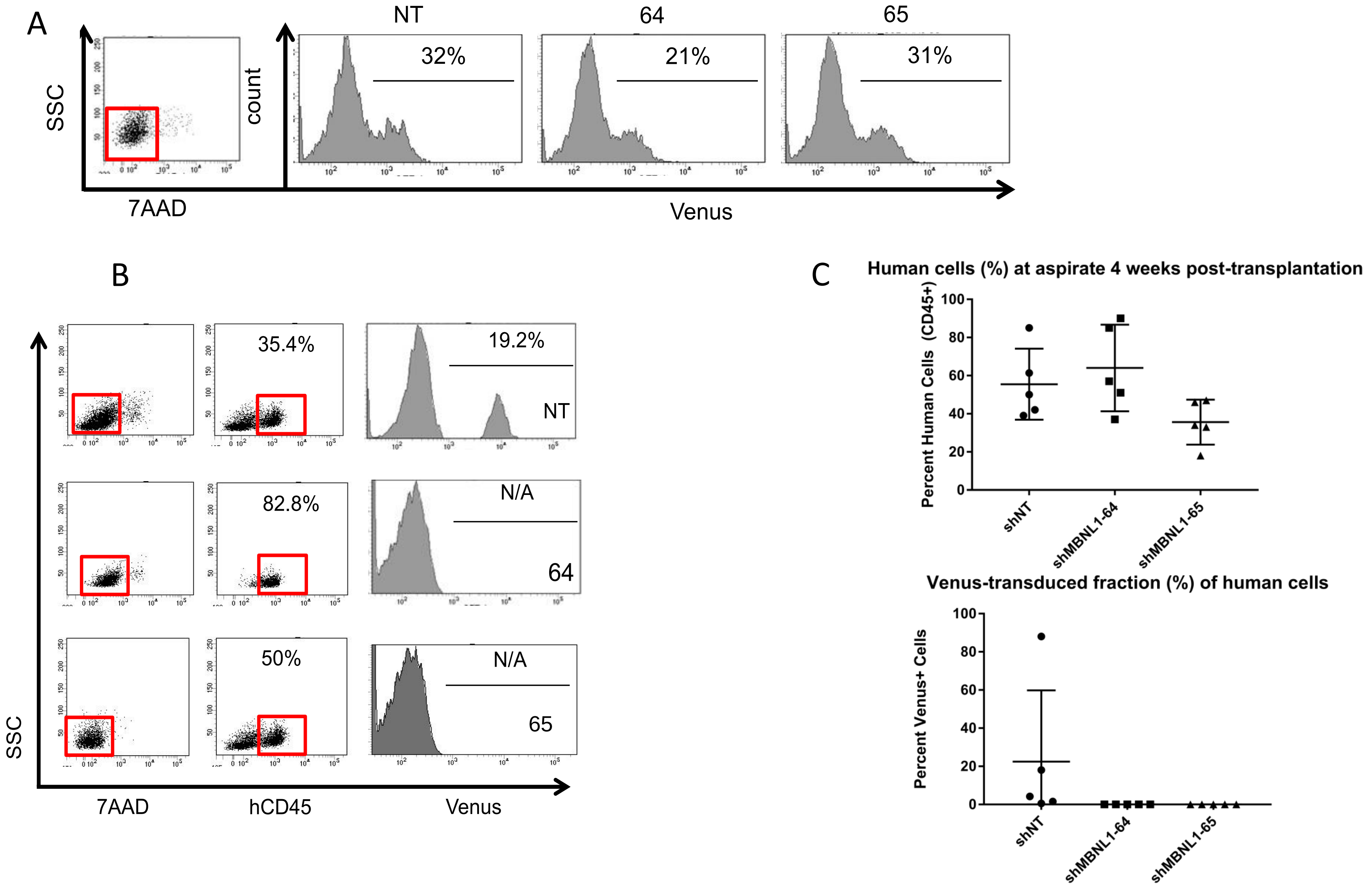

Supplementary Figure 3. *In Vivo MBNL1* knockdown study with MLL-AF9/NRAS^{G12D} cells. (A). MLL-AF9/NRAS^{G12D} human leukemia cells were transduced with *MBNL1*-specific (shMBNL1-64 or shMBNL1-65) or non-targeting (NT) shRNA and transplanted into immunodeficient NSG mice (n=5 mice per group). Histograms show the efficiency of shRNA transduction as determined by Venus expression. **(B)**. Four weeks after transplantation bone marrow aspirates were collected and tested for the presence of human CD45^{+} cells (middle panel) and levels of shRNA-transduced CD45^{+} and Venus-positive cells (right panel). Representative data from each group is shown. **(C).** Graphs summarize the data shown in (B.) from 5 mice in each experimental group. Points show a value in individual mice. Error bars represent mean $\pm$ SD.

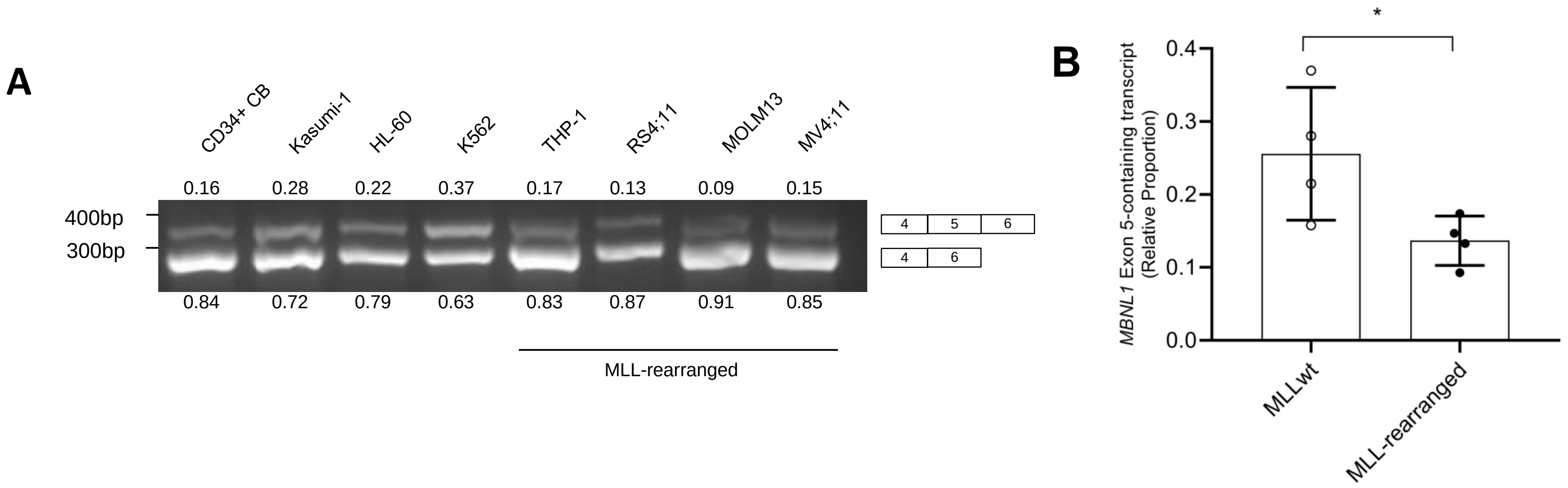

Supplementary Figure 4. Representative RT-PCR analysis of Exon 5 Exclusion/Inclusion in MLL-rearranged vs WT cell lines. (A). Representative qRT-PCR analysis of *MBNL1* transcript including or excluding exon 5. Upper band represents inclusion of *MBNL1* exon 5 in $CD34^+$ cord blood cells and in different human leukemia cell lines that either contain an MLL-fusion gene (THP-1, RS4;11, MOLM13 or MV4;11) or lack an MLL-fusion gene (HL-60, K562, Kasumi-1). **(B)** Comparing relative proportions of exon 5-retaining transcripts shows that MLL-rearranged cell lines (i.e. high MBNL1 expression) have a decreased fraction of retained exon 5, supporting the auto-splicing activity of MBNL1. Error bars represent mean ± SD. *p = 0.0497 by two-tailed unpaired t-test. Representative images/calcuations above of 2 biological replicates; n = 4 cell lines per category.

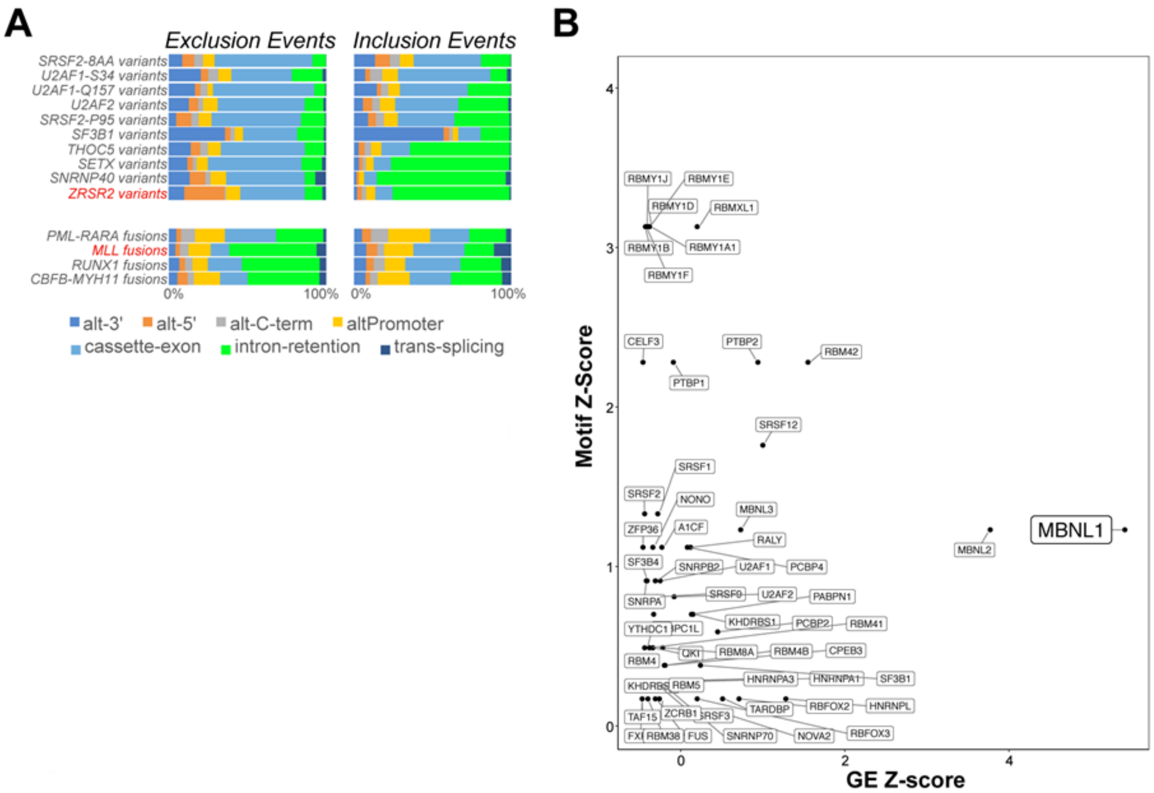

Supplementary Figure 5. Additional Primary Evidence for Repression of Intron-Retention in MLL Mediated by *MBNL1*. A) Relative ratio of alternative exon inclusion (left) and exon inclusion (right) for distinct categories of alternative splicing or alternative promoter regulation in different adult AML subtypes (Leucegene cohort). Alternative splicing events were quantified by MultiPath-PSI as described (Methods). B) Statistical enrichment of RNA recognition elements from the CisBP-RNA database with the software HOMER for alternatively spliced exons/introns (Z-score) and *MBNL1* gene expression in MLL rearranged patient samples versus cytogenetically normal matched AMLs from the Leucegene cohort[7].

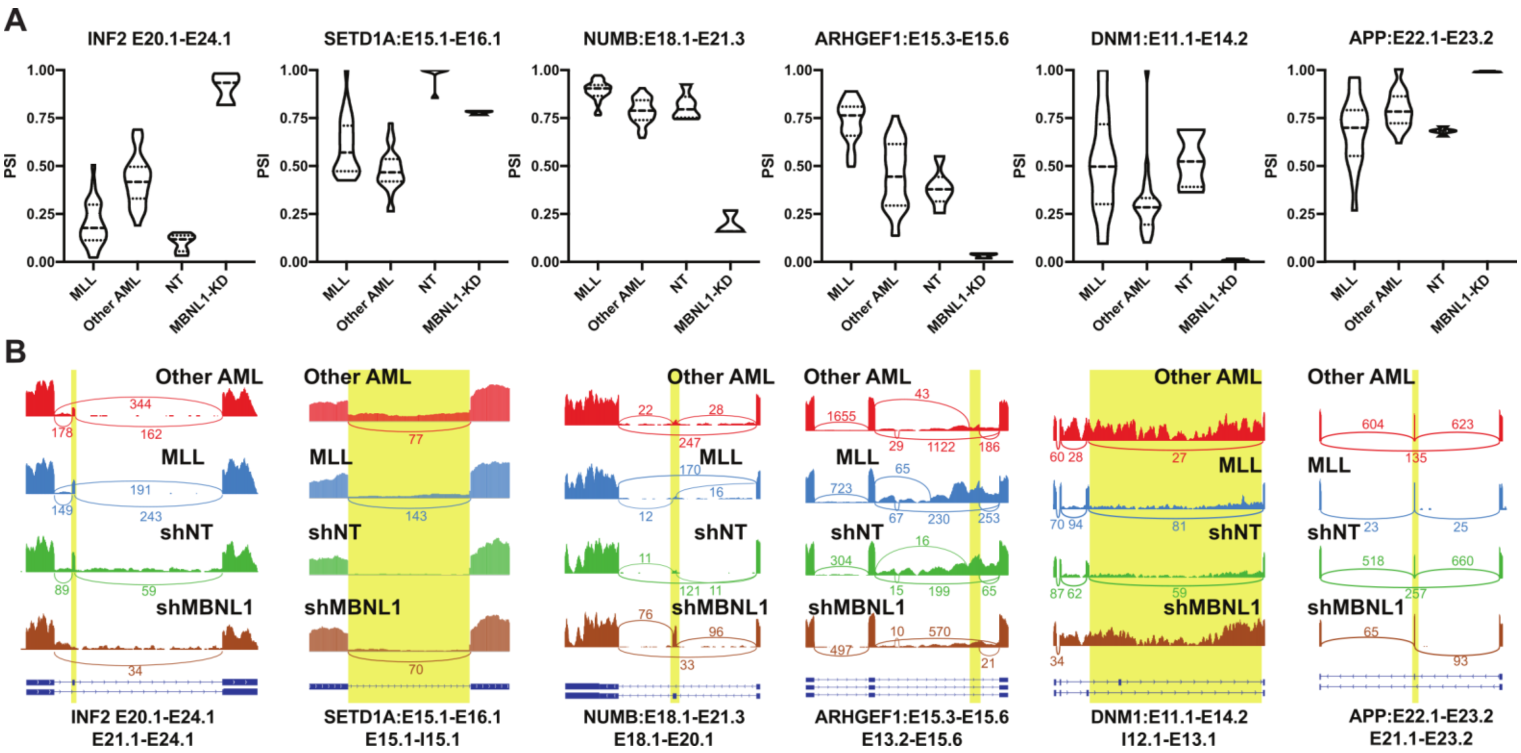

Supplementary Figure 6. *MBNL1*-dependent MLL patient and in vitro alternative splicing events. A) Violin plots displaying the Percent Spliced In (PSI) values for experimental replicates (knockdown) or patients with MLL rearrangements versus cytogenetically normal AMLs (Leucegene). The selected examples demonstrate concordant differential splicing events in both AML patients with and without MLL-oncofusions and those in *MBNL1*-knockdown MOLM13 cells. B) SashimiPlots for the alternative splicing events from panel A, produced in the software IGV. Curved lines indicate exon-exon junctions with RefSeq transcript exon-intron definitions indicated below each plot. Numbers indicate junction read counts. Yellow highlighted regions indicate the predicted alternatively spliced intervals from MultiPath-PSI based on the displayed alternative splicing event annotations (exonic regions from the AltAnalyze Ensembl 72/UCSC hg19 database).

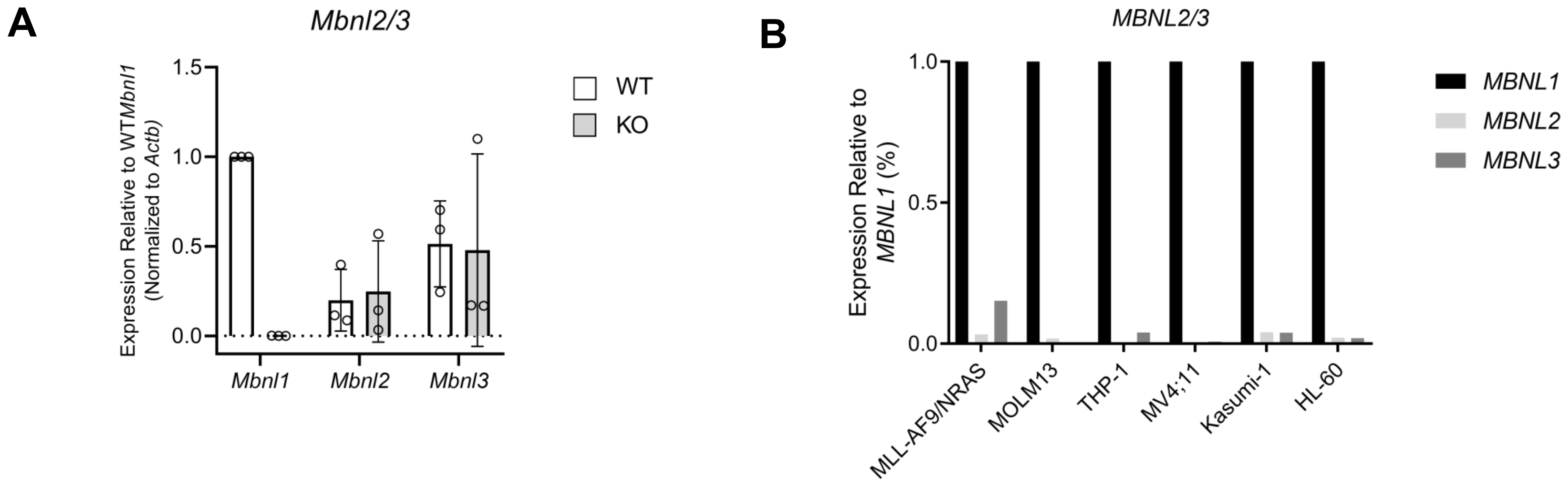

Supplementary Figure 7. *Mbnl2* and *Mbnl3* gene expression in murine and human leukemic cells. (A). qRT-PCR analysis of *Mbnl2* and *Mbnl3* gene expression in murine leukemic cells with and without *Mbnl1* knockout. The graph shows expression relative to *Mbnl1* in WT controls; individual expression levels normalized to actin. Bars show mean +/- SD. Data is from 3 biological replicates. **(B)**. Representative qRT-PCR analysis of *MBNL* family gene expression profile in different human leukemia cell lines. *MBNL2/3* expression is depicted as relative to *MBNL1*; levels normalized to *ACTB.*

Supplementary Figure 8: Flow Cytometry Gating Strategies

Figure 2H: Primary patient xenografts

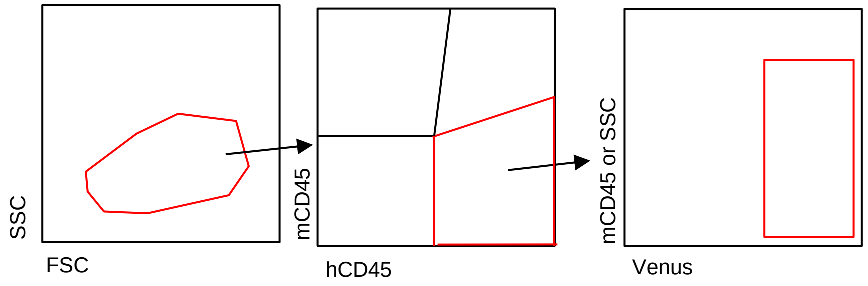

Figure 2I: Apoptosis

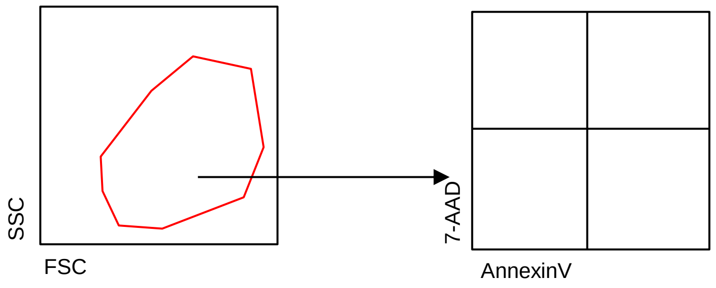

Figure 4B, F: Chimerism

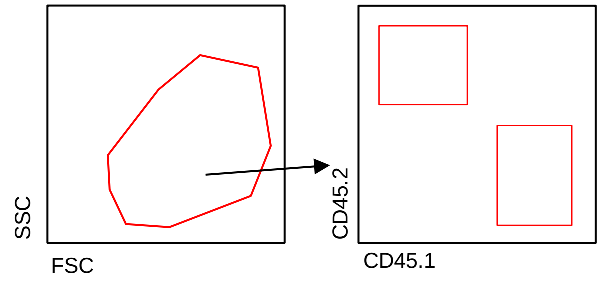

Figure 4E,H: Donor Lineage reconstitution

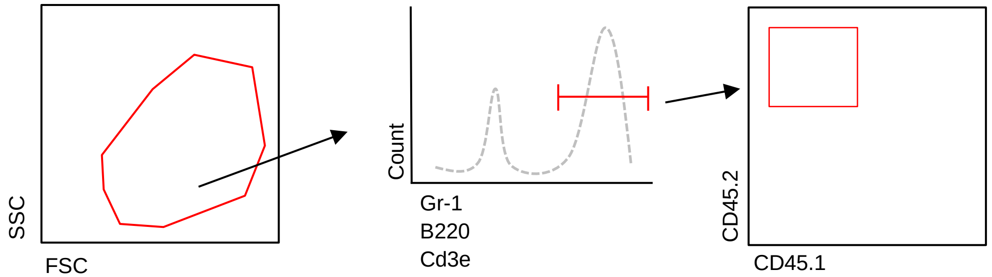

Figure 4J: LSK-SLAM

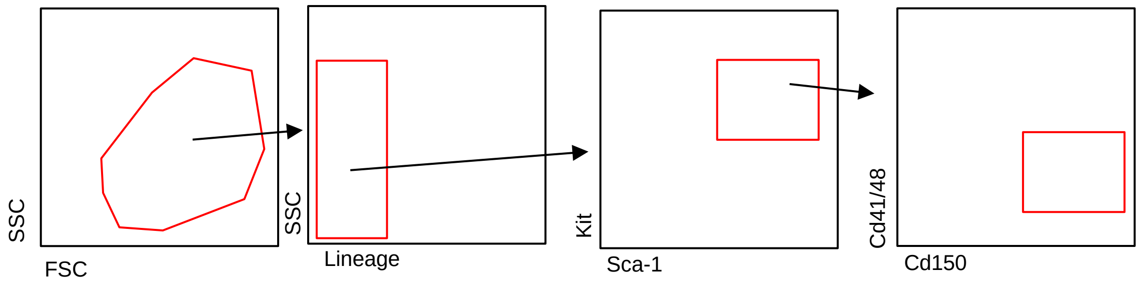

Supplementary References

1 Liu, Y. *et al.* The genomic landscape of pediatric and young adult T-lineage acute lymphoblastic leukemia. *Nat Genet* **49**, 1211-1218, doi:10.1038/ng.3909 (2017).

2 Zhang, J. *et al.* Deregulation of DUX4 and ERG in acute lymphoblastic leukemia. *Nat Genet* **48**, 1481-1489, doi:10.1038/ng.3691 (2016).

3 Andersson, A. K. *et al.* The landscape of somatic mutations in infant MLL-rearranged acute lymphoblastic leukemias. *Nat Genet* **47**, 330-337, doi:10.1038/ng.3230 (2015).

4 Faber, Z. J. *et al.* The genomic landscape of core-binding factor acute myeloid leukemias. *Nat Genet* **48**, 1551-1556, doi:10.1038/ng.3709 (2016).

5 de Rooij, J. D. *et al.* Pediatric non-Down syndrome acute megakaryoblastic leukemia is characterized by distinct genomic subsets with varying outcomes. *Nat Genet* **49**, 451-456, doi:10.1038/ng.3772 (2017).

6 Tyner, J. W. *et al.* Functional genomic landscape of acute myeloid leukaemia. *Nature* **562**, 526-531, doi:10.1038/s41586-018-0623-z (2018).

7 Lavallee, V. P. *et al.* The transcriptomic landscape and directed chemical interrogation of MLL-rearranged acute myeloid leukemias. *Nat Genet* **47**, 1030-1037, doi:10.1038/ng.3371 (2015).
