# Supplementary material for: MBNL1 regulates essential alternative RNA splicing patterns in MLL-rearranged leukemia
Source: Nat Commun. 2020 May 12;11:2369. doi: 10.1038/s41467-020-15733-8 (PMC7217953; doi:10.1038/s41467-020-15733-8)
Supplement: Supplementary file 3 — Description of Additional Supplementary Information [file 41467_2020_15733_MOESM3_ESM.pdf]

Description of Additional Supplementary Files

File Name: Supplementary Data 1
Description: Alternative splicing events identified by MultiPath-PSI from experimental and patient-derived RNAseq dataset comparisons.

File Name: Supplementary Data 2
Description: Concordance analysis between shared alternative splicing events from patient samples, ENCODE, and cell type-specific comparisons identified by MultiPath-PSI.

File Name: Supplementary Data 3
Description: Differentially expressed genes identified by AltAnalyze in MOLM13 experimental conditions.

File Name: Supplementary Data 4
Description: Antibodies used for flow cytometry.

File Name: Supplementary Data 5
Description: RT-PCR primer sequences.
