# Supplementary material for: MBNL1 regulates essential alternative RNA splicing patterns in MLL-rearranged leukemia
Source: Nat Commun. 2020 May 12;11:2369. doi: 10.1038/s41467-020-15733-8 (PMC7217953; doi:10.1038/s41467-020-15733-8)
Supplement: Supplementary file 9 — Reporting Summary [file 41467_2020_15733_MOESM9_ESM.pdf]

nature research

Corresponding author(s): Lynn Lee

Last updated by author(s): Feb 21, 2020

Reporting Summary

Nature Research wishes to improve the reproducibility of the work that we publish. This form provides structure for consistency and transparency in reporting. For further information on Nature Research policies, seeAuthors & Referees and theEditorial Policy Checklist .

Statistics

For all statistical analyses, confirm that the following items are present in the figure legend, table legend, main text, or Methods section.

n/a	Confirmed	
☐	☒	The exact sample size (*n*) for each experimental group/condition, given as a discrete number and unit of measurement
☐	☒	A statement on whether measurements were taken from distinct samples or whether the same sample was measured repeatedly
☐	☒	The statistical test(s) used AND whether they are one- or two-sided<br>*Only common tests should be described solely by name; describe more complex techniques in the Methods section.*
☒	☐	A description of all covariates tested
☒	☐	A description of any assumptions or corrections, such as tests of normality and adjustment for multiple comparisons
☐	☒	A full description of the statistical parameters including central tendency (e.g. means) or other basic estimates (e.g. regression coefficient) AND variation (e.g. standard deviation) or associated estimates of uncertainty (e.g. confidence intervals)
☐	☒	For null hypothesis testing, the test statistic (e.g. *F*, *t*, *r*) with confidence intervals, effect sizes, degrees of freedom and *P* value noted<br>*Give P values as exact values whenever suitable.*
☒	☐	For Bayesian analysis, information on the choice of priors and Markov chain Monte Carlo settings
☒	☐	For hierarchical and complex designs, identification of the appropriate level for tests and full reporting of outcomes
☒	☐	Estimates of effect sizes (e.g. Cohen's *d*, Pearson's *r*), indicating how they were calculated

Our web collection on statistics for biologists contains articles on many of the points above.

Software and code

Policy information about availability of computer code

Data collection	BD FACSDiva Version 8.0 was used to collect flow cytometry data. Image Lab was used for nucleic acid gels obtained with the BioRad ChemiDoc.
Data analysis	MS Excel 2010 and GraphPad Prism Version 8 were used to analyze cell and mouse data. FlowJo Version 10 was used to analyze flow cytometry data. AltAnalyze was used for RNA splicing analysis (STAR 2.7 was used for FASTQ files). Nucleic acid gel densitometry was performed with BioRad ImageLab

For manuscripts utilizing custom algorithms or software that are central to the research but not yet described in published literature, software must be made available to editors/reviewers. We strongly encourage code deposition in a community repository (e.g. GitHub). See the Nature Research guidelines for submitting code & software for further information.

Data

Policy information about availability of data

All manuscripts must include a data availability statement. This statement should provide the following information, where applicable:

- Accession codes, unique identifiers, or web links for publicly available datasets
- A list of figures that have associated raw data
- A description of any restrictions on data availability

RNA-seq data (MOLM13, MV4;11 cells with shNT (non-targeting) versus multiple shMBNL1 knockdown conditions): GSE123441. To access this use reviewer token gvqnoaaazrydfex.

Field-specific reporting

Please select the one below that is the best fit for your research. If you are not sure, read the appropriate sections before making your selection.

- [x] Life sciences
- [ ] Behavioural & social sciences
- [ ] Ecological, evolutionary & environmental sciences

For a reference copy of the document with all sections, see nature.com/documents/nr-reporting-summary-flat.pdf

Life sciences study design

All studies must disclose on these points even when the disclosure is negative.

Sample size	Sample sizes were chosen based on what sample sizes were sufficient in similar experiments done during pilot studies in our lab.
Data exclusions	In the experiment testing persistence of shMBNL1 knockdown primary patient cells in vivo, data from one mouse was excluded given that qRT-PCR analysis revealed lack of MBNL1 knockdown (this is stated in the manuscript).
Replication	Experimental findings were able to be replicated.
Randomization	Cells and mice were randomly allocated into experimental groups.
Blinding	Blinding was not relevant to our study as we were working with datasets, cells, and mouse models. There were no human subjects.

Reporting for specific materials, systems and methods

We require information from authors about some types of materials, experimental systems and methods used in many studies. Here, indicate whether each material, system or method listed is relevant to your study. If you are not sure if a list item applies to your research, read the appropriate section before selecting a response.

Materials & experimental systems

n/a	Involved in the study	
[ ]	[x]	Antibodies
[ ]	[x]	Eukaryotic cell lines
[x]	[ ]	Palaeontology
[ ]	[x]	Animals and other organisms
[x]	[ ]	Human research participants
[x]	[ ]	Clinical data

Methods

n/a	Involved in the study	
[x]	[ ]	ChIP-seq
[ ]	[x]	Flow cytometry
[x]	[ ]	MRI-based neuroimaging

Antibodies

Antibodies used

Mouse monoclonal anti-CD45.1 eFluor450: ThermoFisher; Cat#48-0453-80; RRID: AB_1272225
Mouse monoclonal anti-CD45.2 APC: ThermoFisher; Cat#17-0454-81; RRID: AB_469399
Rat monoclonal anti-mouse Ly-6G APC: ThermoFisher; Cat#17-9668-80; RRID: AB_2573306
Hamster monoclonal anti-mouse CD3e FITC: ThermoFisher; Cat#11-0033-81; RRID: AB_837090
Rat monoclonal anti human/mouse B220 (PE-Cy7): ThermoFisher; Cat#25-0452-82; RRID: AB_469627
Rat monoclonal anti human/mouse B220 (eFlour 450): ThermoFisher; Cat#48-0452-82; RRID: AB_1548761
Rat monoclonal anti-human/mouse B220 (PE): ThermoFisher; Cat#12-0452-82; RRID: AB_465671
Rat monoclonal anti-human/mouse CD11b (PE-Cy5): BioLegend; Cat#101209; RRID: AB_312792
Mouse monoclonal anti-human CD45 (APC): BD Biosciences; Cat#555485; RRID: AB_1645479
Mouse monoclonal anti-human CD45 (PE). BD Biosciences; Cat#555483; RRID: AB_2033960
APC Annexin V: BD Biosciences; Cat#550474; RRID: AB_2034024
Rat monoclonal anti-mouse CD150 (APC): ThermoFisher/eBioscience; Cat# 17-1502-80, RRID:AB_1582223
Rat monoclonal anti-mouse CD41a (FITC): ThermoFisher/eBioscience; Cat# 11-0411-81, RRID:AB_763482
Hamster monoclonal anti-mouse CD48 (FITC): ThermoFisher/eBioscience; Cat# 11-0481-81, RRID:AB_465076
Rat monoclonal anti-mouse CD117 (PE): BD Biosciences Cat# 553355, RRID:AB_394806
Rat monoclonal anti-mouse Sca-1 (PerCP-Cy5.5): ThermoFisher/eBioscience; Cat# 45-5981-80, RRID:AB_914370
eFluor450 streptavidin: ThermoFisher/eBioscience; Cat# 48-4317-82, RRID:AB_10359737
Normal Mouse polyclonal anti-IgG: Millipore Sigma; Cat#12-371; RRID: AB_145840
Rabbit polyclonal anti-human MLL1: Bethyl; Cat#A300-086A; RRID: AB_242510
Mouse monoclonal anti-human MBNL1: Millipore Sigma; Cat#MABE70; RRID: AB_10808499
Rabbit monoclonal anti-human DOT1L: Cell Signaling Technology; Cat#77087S
Rabbit monoclonal anti-human SETD1A: Cell Signaling Technology; Cat#61702S

	Rabbit monoclonal anti-human Actin: Cell Signaling Technology; Cat#4970; RRID: AB_2223172<br>Rabbit monoclonal anti-human β-tubulin: Cell Signaling Technology; Cat#2128S; RRID: AB_823664<br>Rabbit monoclonal anti-human Lamin B1: Cell Signaling Technology; Cat#12586; AB_2650517
Validation	All antibodies were purchased from commercial manufacturers. Validation and citation information can be found on the manufacturer's website.

Eukaryotic cell lines

Policy information about cell lines

Cell line source(s)	The following cell lines were obtained from DSMZ: MOLM13. RCH-ACV<br>The following cell lines were obtained from ATCC: RS4;11, Kasumi-1, MV4;11, K562, HL60, Molt-4, THP-1
Authentication	Cell lines were periodically validated by STR genotyping through Genetica Cell Line Testing (LabCorp).
Mycoplasma contamination	Cells were tested and were negative for mycoplasma contamination.
Commonly misidentified lines (See ICLAC register)	None of the cell lines utilized in this study are recognized by the ICLAC as being commonly misidentified.

Animals and other organisms

Policy information about studies involving animals; ARRIVE guidelines recommended for reporting animal research

Laboratory animals	All animals used for this study were 6-12 weeks old.<br>Mouse: donor MBNL1-/-: C57BL/6<br>Mouse: recipient B6.SJL-Ptprca Pepcb/BoyJ<br>Mouse: NOD/LtSz-SCID interleukin-2(IL2)RG-/- (NSG)<br>Mouse: NOD/LtSz-SCID interleukin-2(IL2)RG-/- SGM3 (NSGS)
Wild animals	The study did not involve wild animals.
Field-collected samples	The study did not involve samples collected from the field.
Ethics oversight	All animal experiments were carried out in accordance with the guidelines of the Institutional Animal Care and Use Committee (IACUC).

Note that full information on the approval of the study protocol must also be provided in the manuscript.

Flow Cytometry

Plots

Confirm that:

- [x] The axis labels state the marker and fluorochrome used (e.g. CD4-FITC).
- [x] The axis scales are clearly visible. Include numbers along axes only for bottom left plot of group (a 'group' is an analysis of identical markers).
- [x] All plots are contour plots with outliers or pseudocolor plots.
- [x] A numerical value for number of cells or percentage (with statistics) is provided.

Methodology

Sample preparation	See method section, paragraph "Flow cytometry"
Instrument	BD FACSCanto II
Software	BD FACSDiva Version 8.0 was used to collect flow cytometry data. FlowJo Version 10 was used to analyze flow cytometry data.
Cell population abundance	See method section, paragraph "Flow cytometry." At least 10,000 events were collected.
Gating strategy	Cells were gated using the FSC-A vs. SSC-A plot to exclude dead cells and cell debris. Background fluorescence was defined using mock control/control cells (compensation controls were created and compensation was calculated on BD FACSDiva Version 8.0 software).

- [x] Tick this box to confirm that a figure exemplifying the gating strategy is provided in the Supplementary Information.
